# Techniques, Timing & Prognosis of Post Infarct Ventricular Septal Repair: a Re-look at Old Dogmas

**DOI:** 10.21470/1678-9741-2016-0032

**Published:** 2017

**Authors:** Amber Malhotra, Kartik Patel, Pranav Sharma, Vivek Wadhawa, Tarun Madan, Jagdish Khandeparkar, Komal Shah, Sanjay Patel

**Affiliations:** 1 Department of Cardiovascular and Thoracic Surgery of the U. N. Mehta Institute of Cardiology and Research Center (affiliated to BJ Medical College, Ahmedabad), Gujarat, India.; 2 Department of Cardiology of the U. N. Mehta Institute of Cardiology and Research Center (affiliated to BJ Medical College, Ahmedabad), Gujarat, India.; 3 Department of Research of the U. N. Mehta Institute of Cardiology and Research Center (affiliated to BJ Medical College, Ahmedabad), Gujarat, India.

**Keywords:** Myocardial Infarction, Heart Septal Defects, Ventricular, Cardiac Surgical Procedures, Prognosis

## Abstract

**Objective:**

The study aimed to identify the factors affecting the prognosis of post myocardial infarction (MI) ventricular septal rupture (VSR) and to develop a protocol for its management.

**Methods:**

This was a single center, retrospective-prospective study (2009-2014), involving 55 patients with post MI VSR. The strengths of association between risk factors and prognosis were assessed using multivariate logistic regression analysis. The UNM Post MI VSR management and prognosis scoring systems (UPMS & UPPS) were developed.

**Results:**

Thirty-day mortality was 52.5% (35% in the last 3 years). Twenty-eight (70%) patients underwent concomitant coronary artery bypass grafting. Residual ventricular septal defect was found in 3 (7.5%) patients. The multivariate analysis showed low mean blood pressure with intra-aortic balloon pump (OR 11.43, *P*=0.001), higher EuroSCORE II (OR 7.47, *P*=0.006), higher Killip class (OR 27.95, *P*=0.00), and shorter intervals between MI and VSR (OR 7.90, *P*=0.005) as well as VSR and Surgery (OR 5.76, *P*=0.016) to be strong predictors of mortality. Concomitant coronary artery bypass grafting (*P*=0.17) and location (*P*=0.25) of VSR did not affect the outcome. Mean follow-up was 635.8±472.5 days and 17 out of 19 discharged patients were in NYHA class I-II.

**Conclusion:**

The UNM Post-MI VSR Scoring Systems (UPMS & UPPS) help in management and prognosis, respectively. They divide patients into 3 groups: 1) Immediate Surgery - Patients with scores of <25 require immediate surgery, preferably with extracorporeal membrane oxygenation support, and have poor prognosis; 2) Those with scores of 25-75 should be managed with "Optimal Delay" and they have intermediate outcomes; 3) Patients with scores of >75 can undergo Elective Repair and they are likely to have good outcomes.

**Table:** 

Abbreviations, acronyms & symbols				
ACE	= Angiotensin-converting-enzyme inhibitor		MI	= Myocardial infarction
AUC	= Area under the curve		NYHA	= New York Heart Association
CABG	= Coronary artery bypass grafting		PLV	= Posterior left ventricular artery
CI	= Confidence intervals		PTFE	= Polytetrafluoroethylene
DVD	= Double vessel disease		RCA	= Right coronary artery
ECMO	= Extracorporeal membrane oxygenation		ROC	= Receiver operating characteristic
IABP	= Intra-aortic balloon pump		SD	= Standard deviation
ICU	= Intensive care unit		TEE	= Transesophageal echocardiography
LAD	= Left anterior descending		TVD	= Triple vessel disease
LVEF	= Left ventricular ejection fraction		VSR	= Ventricular septal rupture

## INTRODUCTION

In spite of advances in surgical techniques, surgical repair of post myocardial infarction (MI) ventricular septal rupture (VSR) (post-MI VSR) is still associated with a high mortality rate of 20%-50%^[1-3]^. In unoperated cases, early death is common; 50% die in <7 days, 70% within 2 weeks, and 80%-90% die within 4 weeks^[4-7]^.

The first surgical repair of post-MI VSR was performed by Cooley et al.^[8]^, in 1957, followed by documentation of several other series^[9,10]^. However, the available literature is very scant and scattered due to the rarity, discrepancy in clinical presentation, and complex natural history of the condition^[9]^. Timing of surgery and associated co-morbidities play an important role in determining the outcome of patients^[10]^. In recent times, due to advances in interventional cardiology, extracorporeal membrane oxygenation (ECMO) and intensive care unit (ICU) care, a multitude of options have become available to the 'heart team' to do either an immediate surgery or delay/defer surgery. However, objective guidelines for proper management of this entity are still not available due to the relative rarity of this highly fatal condition.

We present our center's experience with 55 patients with post-MI VSR and we suggest the use of the UNM Post-MI VSR Scoring Systems to guide management and indicate prognosis of patients.

## METHODS

Between January 2009 and January 2014, 55 patients were diagnosed with post-MI VSR and 40 of them (26 male; 14 female), who underwent surgery in the Department of Cardiothoracic Surgery, U.N. Mehta Institute of Cardiology & Research Center, were included in our study. Ten patients who did not give consent for surgery were managed medically. The remaining five patients underwent elective device closure since they presented VSR later than 4 weeks after MI, were in New York Heart Association (NYHA) I/II class, and did not require coronary artery bypass grafting (CABG). After approval from the ethics committee, perioperative data were collected from hospital records and written informed consent of the living patients was obtained for follow-up. Data for the following variables were collected: age, gender, time between MI and VSR, urgency of operation, prior cardiac surgery, history of previous (>3 months) MI, smoking, diabetes, hypertension, respiratory disease, renal dysfunction, congestive cardiac failure, cardiogenic shock, intra-aortic balloon pump (IABP) usage, mean blood pressure before and after IABP insertion, inotrope usage, ventilatory support, EuroSCORE II, Killip class, extent of coronary disease, and left ventricular ejection fraction (LVEF). The outcomes measured for our study were: hospital mortality, length of ICU stay, post-operative hospital stay, stroke, peri- operative MI, re-operation for bleeding, renal failure, and need for tracheostomy. We also noted the incidence of arrhythmias and duration of post-operative inotropes and IABP support.

### Surgical Technique

After complete cardiological assessment using echocardiography and coronary angiography, the patients were approached through a median sternotomy. IABP was instituted in all patients (if not inserted in the pre-operative period) just after induction of anesthesia. After cardiopulmonary bypass was established using aortic and bicaval cannulation, the aorta was cross-clamped and antegrade blood cardioplegia and intermittent retrograde cardioplegia were used in all cases. If CABG was required, distal anastomoses were made first. A vacuum positioner system was used to expose the posterior VSRs. The septal rupture was approached through the infarcted area of the myocardium, preferably through the left ventricle. A variant of the Daggett's technique was used^[11]^. Infarctectomy was not routinely performed, but care was taken to go at least 1 cm beyond the infarcted margin. All the anterior and most of the posterior defects were closed by a 0.6 mm polytetrafluoroethylene (PTFE) patch fixed to the non-infarcted region of the septum with interrupted horizontal pledgeted 4-0 polypropylene mattress suture (26 mm needle), keeping the pledget on right ventricular side. Deep bites extending into healthy myocardium were taken to prevent sutures from cutting through. Infarct exclusion technique was used when margins were too friable or healthy area was indistinguishable from the infarcted area. The ventriculotomy was closed using 4-0 polypropylene sutures (26 mm needle) in interrupted and continuous fashion with PTFE felt strips on each side of the ventriculotomy, as described by Daggett et al.^[11]^. After, cardiopulmonary bypass was discontinued, oxygen saturation of right atrial and pulmonary arterial blood was measured along with transesophageal echocardiography (TEE) to rule out residual ventricular septal defect. IABP and inodilators (levosimendan/milrinone) were used to stabilize the patient in the immediate postoperative period. Inotropes were guided by cardiac output measured through the Swan Ganz catheter. All patients were started on enteral nutrition through nasogastric tube from postoperative day 1. Patients were routinely ventilated till inotropic requirement was high. Patients were weaned off from the ventilator once inotropic requirement became reasonable (vasoactive inotropic score of <10)^[12]^ and respiratory parameters were satisfactory. IABP continued to be used for at least 4-5 days postoperatively and then it was gradually tapered off once inotropic support decreased to a minimal dose (vasoactive score of ≤5). Inodilators were tapered 1 or 2 days after IABP removal. Postoperatively, patients were given amiodarone, antiplatelets, statins, beta blockers, spironolactone, and angiotensin-converting-enzyme inhibitor (ACE) inhibitors. Amiodarone was discontinued after 15 days of surgery.

### Patient Follow-up

Late mortality, NYHA class, and residual defects were noted on follow-up. Patients were followed up in an outpatient setting and/or by telephonic interview. Echocardiography was performed at three-month intervals for the first 6 months, and then at 6-month intervals thereafter. Preoperative echo parameters were compared with those at follow-up. Residual defects, if any, were analyzed. Presence or absence of mitral regurgitation, right and left ventricular function, and pulmonary artery pressures were also analyzed at follow-up echocardiography.

### Statistical Analysis

The statistical calculations were performed using SPSS software v 20.0 (Chicago, IL, USA). Continuous data were expressed as mean ± standard deviation (SD). Patient survival rates were calculated using Kaplan-Meier. Univariate analysis of continuous data was performed using Student's t-test, whereas chi-square test was used for the categorical data. Multivariate logistic regression was used to estimate independent risk factors for the factors with a significant *P* value (<0.05) on univariate analysis. An attempt has been made to develop a deterministic quantitative risk score in order to assess the risk involved with patient's clinical condition. Factors for the risk score calculation were screened by a univariate logistic screening model. Furthermore, these clinical factors were used in risk score calculations. Theoretical details for the index can be given as follows. The risk score is comprised of two elements: weightage {statistical weightage (as provided by regression analysis) of factors thought to be important as per surgical literature} and value of the clinical parameter. Let '***X_i_***' be the value of the corresponding clinical variable and '***W_i_***' be the combined weightage of the corresponding variable. Thus, the proposed risk score is a linear function of clinical parameters given as below:

$$\mathrm{Risk}\;\mathrm{Factor}=\sum W_{i}x_{i}$$

To assess the performance of the risk score with respect to predicting survival, a receiver operating characteristic (ROC) curve was plotted for the risk scores. The area under the curve (AUC) and 95% confidence intervals (CI) were then estimated. Various cut-off scores were calculated and analyzed for survival.

## RESULTS

### Patient Characteristics

Sixty-five percent of the patients undergoing surgical post-MI VSR repair were male and 35% were female. Mean age of the patients was 61.65±7.6 years (Table 1). The most common coronary arteries involved were the left anterior descending (LAD) at 75% and the right coronary artery (RCA) at 47.5%. Other demographic characteristics are mentioned in Table 1.

**Table 1:** Univariate analysis of risk factors.

	Survival n= 19	Death n=21	*P* Value
Age (years; Mean ± SD)	60.68±5.6	62.52±9.0	0.452
Gender (Male)	16 (84.2%)	10 (47.6%)	0.112
**History**			
Hypertension (n; %)	9 (47.4%)	12 (57.1%)	0.752
Diabetes (n; %)	6 (31.6%)	10 (47.6%)	0.349
Smoking (n; %)	8 (42.1%)	5 (23.8%)	0.314
Stroke (n; %)	1 (5.3%)	2 (9.5%)	1.000
Previous MI (n; %)	3 (15.8%)	4 (19%)	1.000
NYHA Class (years; Mean±SD)	3.11±0.5	3.5±0.5	0.036
**Preoperative Angiography**			
Single Vessel Disease (SVD) (n; %)	11 (57.9%)	7 (33.3%)	0.262
Double Vessel Disease (DVD) (n; %)	6 (31.6%)	9 (42.9%)	0.252
Triple Vessel Disease (TVD) (n; %)	2 (10.5%)	5 (23.8%)	0.111
**Culprit Vessel**			
Anterior VSR (n; %)	14 (73.6%)	13 (61.9%)	0.262
Posterior VSR (n; %)	5 (26.3%)	8 (38.1%)	0.256
EuroSCORE II (Mean±SD)	17.26±8.1	26.02±8.8	0.002
Killip class (Mean±SD)	2.84±0.3	3.98±0.3	0.000
Pre EF (%; Mean±SD)	37.10±7.3	36.90±7.8	0.934
Post EF (%; Mean ± SD)	39.73±6.7	35.47±8.3	0.086
Mean blood pressure after IABP insertion (mmHg; Mean±SD)	104.95±12.3	96.85±9.9	0.027
VSR Diameter (mm; Mean±SD)	12.52±7.0	12.54±5	0.992
AOX Time (Min; Mean±SD)	101.35±28.2	109.73±21.1	0.318
CPB Time (Min; Mean±SD)	149.11±46.7	168.05±43.9	0.219
Concomitant CABG done (n; %)	11 (57.8%)	17 (80.9%)	0.170
Time between MI and Surgery (Days; Mean±SD)	8.63 ±4.3	4.24±2.4	0.000
Time between Admission and Surgery (Days; Mean±SD)	4.16 ±3.3	2.90±3.7	0.273
Time between IABP and Surgery (Days; Mean±SD)	3.79±2.8	2.38±1.8	0.069
Time between MI and VSR (Days; Mean±SD)	4.16±1.9	2.29±1.4	0.001
Time between VSR and Surgery (Days; Mean±SD)	4.47±3.5	1.90±1.3	0.004
**Postoperative Complications**			
Renal Failure (n; %)	4 (21.05%)	4 (19.04%)	0.8123
Tracheostomy (n; %)	-	2 (9.5%)	0.5133
Postoperative Stroke (n; %)	1 (5.2%)	1 (4.7%)	0.5133
Residual VSD (n; %)	2 (10.52%)	1 (4.76%)	0.9282
Postoperative Bleeding (n; %)	1 (5.26%)	2 (9.52%)	0.9282
Emergency (n; %)	15 (78.9%)	19 (90.4%)	0.5644

NYHA=New York Heart Association; LAD=left anterior descending artery; RCA=right coronary artery; VSR=ventricular septal rupture; AOX=aortic cross clamp; CPB=cardiopulmonary bypass; CABG=coronary artery bypass grafting; EF=ejection fraction; LIMA=left internal mammary artery; VSD=ventricular septal defect; MI=myocardial infarction; IABP=intra-aortic balloon pump

### Mortality Statistics and Contributing Risk Factors

**Fig. 1:**
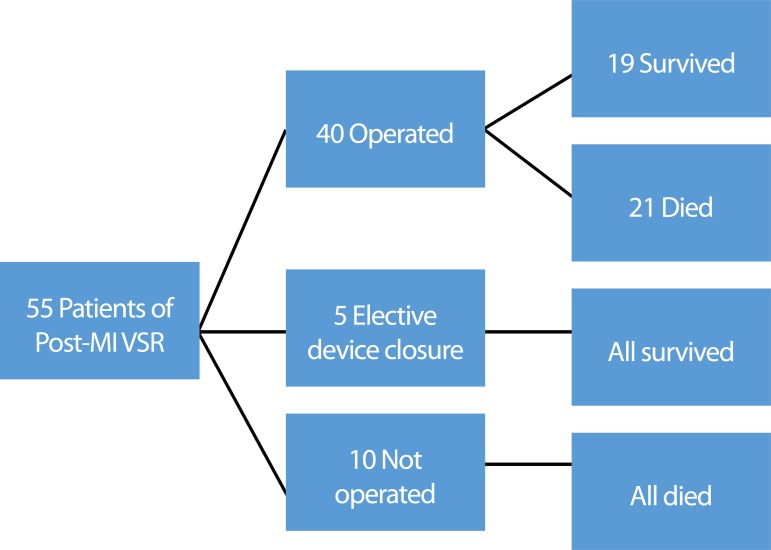
Flow chart showing different interventions and outcomes of patients.

Out of the 55 patients, all ten who did not undergo surgery died. All five patients who underwent elective device closure of VSR after 4-6 weeks survived. The 30-day hospital mortality in the surgical group was 52.5% (21 patients), where two (9.5%) deaths were intraoperative and 19 (90.5%) deaths occurred in the intensive care unit (Figure 1). The two patients who died in the operation room had an inferior wall MI with posterior VSR. One patient could not be weaned off bypass while the other patient died due to uncontrolled bleeding. In the remaining 19 patients, the most common cause of death was low cardiac output in 15 (71.5%) cases, followed by sepsis in the remaining 4 (19%) cases.

It was observed that patients who died had lower mean blood pressure after IABP insertion (96.85±9.9 *vs.* 104.95±12.3, *P*=0.027), higher NYHA class (3.5±0.5 *vs.* 3.1±0.5, *P*=0.036), higher EuroSCORE-II (26.02±8.8 *vs.* 17.26±8.1, *P*=002), higher Killip class (3.98±0.3 *vs.* 2.84±0.3, *P*=0.00) and shorter time interval between occurrence of VSR after MI (2.29±1.4 *vs.* 4.16±1.9, *P*=0.001) than those who survived. The successful and safe postponement of surgery after MI (*P*=0.00) and VSR (*P*=0.004) occurrence was also found to favor good surgical outcomes (Table 1). History of hypertension, diabetes, stroke, and previous MI was higher in the mortality group compared to the survival group, however, those differences did not reach a statistically significant level [(*P*>0.05), Table 1]. Likewise, the severity of coronary artery disease, VSR location, preoperative LVEF, and concurrent CABG had no statistically significant influence on mortality (Table 1). On the logistic regression, we found that low mean blood pressure after IABP insertion (OR 11.43, *P*=0.001), higher EuroSCORE II, (OR 7.47, *P*=0.006), higher Killip class (OR 27.95, *P*=0.00), shorter intervals between MI and VSR (OR 7.90, *P*=0.005) and VSR and Surgery (OR 5.76, *P*=0.016) were independent predictors of mortality (Table 2).

**Table 2:** Regression analysis (odds ratio).

			95% C.I.	
Variables	EXP(B)	Sig.	Lower	Upper
Mean blood pressure after IABP	11.435	0.001	8.780	22.68
Killip's score	27.948	0.000	20.230	38.91
EuroSCORE II	7.470	0.006	2.680	17.13
Time between MI and VSR	7.906	0.005	5.300	19.19
Time between VSR and Surgery	5.761	0.016	1.920	11.23

ABP=intra-aortic balloon pump; MI=myocardial infarction; VSR=ventricular septal rupture

In the studied population, a residual defect was noted in 3 (7.5%) cases. One patient was reoperated and died in the postoperative period while the other two had hemodynamically insignificant VSDs which were managed conservatively. They continued to be in NYHA-II until the last follow-up. There was no statistically significant relationship between location of VSR and occurrence of residual defect (*P*=0.53).

**Fig. 2:**
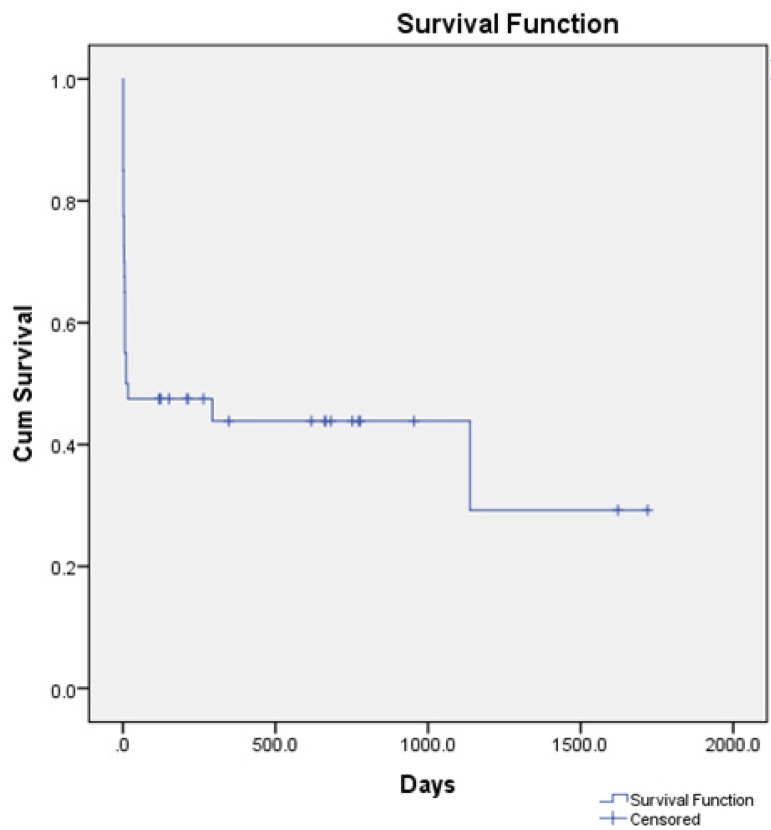
Kaplan Meier Curve of survivors.

Two out of 19 patients discharged from the hospital died during follow-up. Mean follow-up time was 635.8±472.5 (range 118-1720) days. Cause of death was unknown. All surviving patients were in NYHA class I-II at the last follow-up. The survival analysis is shown as Kaplan-Meier survival function (Figure 2).

### UNM-MI VSR Scoring Systems for Management and Prognosis

We developed scoring systems to manage and prognosticate post-MI VSR patients by studying the factors which could affect the outcome using a multivariate logistic screening model (Table 3). The score developed from factors (at the time of presentation of patient) mentioned in Table 3A was described as "UNM Post-MI VSR Management Score" (UPMS). After a patient had been taken up for intervention, according to the management protocol of our institute, the addition of one factor (time from VSR to surgery) gave us the "UNM Post-MI VSR Prognosis Score" (UPPS). The optimum cut-off UPPS was found to be 65. Patients with a UPPS above 65 have a better chance of survival than those having a lower score.

**Table 3A:** UNM Post-MI VSR Management Scoring System.

Factors	Weightage (W)
Mean Blood Pressure -50 in mmHg after IABP	2.5
Killip's score**	-3
EuroSCORE II**	-1
Time between MI and VSR (days):	2

** These factors correlated negatively with survival. IABP=intra-aortic balloon pump; MI=myocardial infarction; VSR=ventricular septal rupture (*Risk Factor=ΣW_i_x_i_*; where, 'W' is Weightage and 'x' is the value of clinical variable).

For example, a 45-year-old male patient who had 65 mmHg mean  blood pressure at presentation and 60 mmHg blood pressure after institution of IABP. He was in Killip class 4, with EuroSCORE II of 10, and it took 3 days from MI to VSR. Score will be {2.5× (60-50)}-{(1 ×10)-(3×4) + (2×3)} = 9. This suggests that this patient requires immediate surgery.

**Table 3B:** UNM Post-MI VSR Prognosis Scoring System for predicting survival in post-MI VSR.

Factors			Weightage (W)
Mean Blood Pressure -50 in mmHg after IABP:			2.5
Killip's score:**			-3
EuroSCORE II:**			-1
Time between MI and VSR (days):			2
Time between VSR and Surgery (days):			1.5
Optimum cut-off: 65			
Score range (n)	Survival (n, %)	Mortality (n, %)	
<25 (9)	-	9 (100%)	
25-50 (10)	3 (30%)	7 (70%)	
50-75 (14)	9 (64.29%)	5 (35.7%)	
>75 (7)	7 (100%)	-	

** These factors correlated negatively with survival. IABP=intra-aortic balloon pump; MI=myocardial infarction; VSR=ventricular septal rupture (*Risk Factor=ΣW_i_x_i_*; where, 'W' is Weightage and 'x' is the value of clinical variable).

For example, in previous cases, if two days are added to the time from VSR to Surgery, the score will be 9+(2×1.5) = 12. This suggests an expected survival of <10%.

**Fig. 3:**
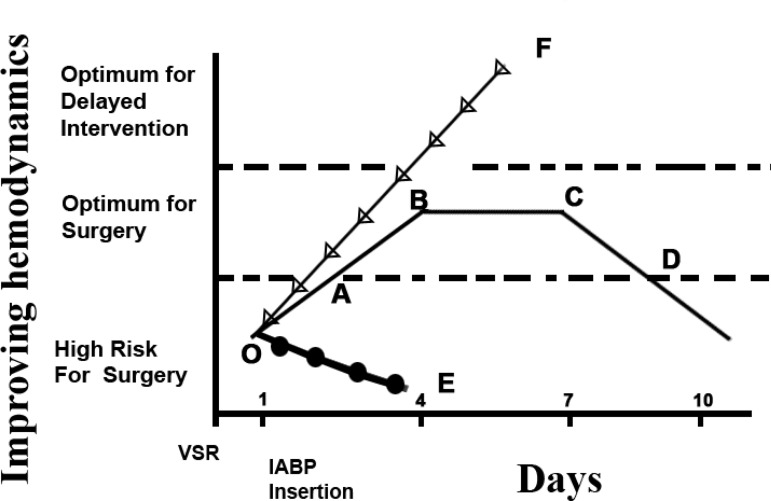
Hemodynamic patterns after IABP institution and suggested timing of intervention. Group 1 (OE Segment, n=18): Patients who were not improving hemodynamically, even after IABP, high inotropic supports and ventilatory support, represented a true surgical emergency and needed Immediate Surgery. Group 2 (OF Segment, n=5): Few patients who were stable, with no clinical deterioration, underwent Elective Repair/Percutaneous Closure after 4-6 weeks. Group 3 (OD Segment, n=22): In this category, patients usually improved hemodynamically with institution of IABP, inotropic support, and with/without ventilatory support. The improvement brought them to lower Killip Class (Segment AB), which plateaued afterwards (Segment BC). After this phase, deterioration started (Segment CD) by virtue of infection and vascular complications. This period (Segment BC), in which patient’s condition was optimum, was the window of opportunity for the surgeon. (Optimal Delay)

While calculating the results of patients operated at different intervals after VSR, three trends were observed (Figure 3).

## DISCUSSION

Post-MI VSR occurs only in 0.2% of patients with acute MI and it was first described by Latham^[13]^ in 1846. In the current study, we have reviewed our experience of 55 patients with post-MI VSR within a span of 5 years. Ten patients were either not referred for surgery or refused surgery. This phenomenon has been documented by Labrousse et al.^[2]^ and GUSTO-I trial^[4]^, which suggest that cardiologists themselves decide about suitability of patients for surgery, leading to fewer patients being referred for surgery. In developing countries, financial constraints also come into picture, further aggravating the situation. Ideally, those patients should be evaluated by a "heart team" consisting of at least one cardiologist, one cardiac surgeon, and one critical care expert.

All patients who were not operated eventually died. Our overall postoperative mortality was 52.5%. However, considering the fact that patients who underwent device closure (n=5) could also have been managed surgically with almost 100% survival (as also seen in our study, all patients with score > 75 survived). This could have resulted in a much lower mortality for our surgical series. Mortality has also been decreasing progressively with better understanding of the condition, 'optimum delay' in surgery after occurrence of VSR, better cardiopulmonary bypass management, improved postoperative care, and extended postoperative IABP usage. Our mortality rate in the first 2 years (2009-2011) was 70% (14/20), decreasing to 35% (7/20) in the last three years (2011-2014). To the best of our knowledge, this is the first study reporting an experience of 55 (40 operated + 5 elective device closure + 10 unoperated) patients within a time span of 5 years from a single center. Higher incidence of post-MI VSR in developing countries could be due to unavailability/delay in primary revascularization or thrombolysis of patients with acute MI.

We found post-MI VSR to be more common in males, especially in those having hypertension. This finding is similar to that observed by Arnaoutakis et al. ^[14]^. But it is contradictory to the reports from GUSTO-1^[4]^, Barker et al.^[9]^, and Serpytis et al.^[15]^ who found female gender to be a risk factor for the development of VSR. We also found that single vessel disease (45%) was the most common type of coronary artery disease associated with post-MI VSR, followed by double vessel disease (37.5%). Similar findings have also been reported by others^[7,15-17]^.

Regarding clinical status, all authors found higher mortality in patients with preoperative cardiogenic shock^[1-3,7,8,14,18]^. In our series, lower mean blood pressure after IABP insertion was found to be an independent risk factor for mortality. We have not found any effect of LVEF on immediate outcome of patients, while Huang et al.^[19]^ found preserved LVEF to have a beneficial effect on early outcomes. In patients with post-MI VSR, LVEF can be fallaciously high due to the presence of a left to right shunt. Therefore, in those patients, pulmonary blood flow/systemic blood flow (Qp/Qs), and pulmonary artery pressures should always be measured to estimate the exact degree of myocardial dysfunction.

The prevalence of triple vessel disease and double vessel disease was higher in the mortality group compared to the survivor group, however, the difference was not statistically significant (*P*=0.11, 0.25). Thus, the number of coronary vessels involved appears to have no significant effect on immediate surgical outcome of the patients. Similar observations were made by Labrousse et al.^[2]^. Unlike others, we did not find any protective effect of concomitant CABG in terms of early mortality^[2,7,14,19,20]^. During follow-up, there was no significant difference in postoperative ejection fraction and NYHA status between patients with concurrent CABG and those without CABG (37.7±6.01 *vs.* 42.5±7.05). This probably reflects the presence of SVD in most surviving patients.

Jeppsson et al.^[1]^ and Dalrymple-Hay et al.^[20]^ attributed the high mortality rates in posterior VSRs to the difficulty of operative exposure and associated right ventricular infarction. Posterior VSRs associated with right ventricular infarction have worse prognosis as compared to posterior VSRs with left ventricular infarction or anterior VSRs. In our study, these differences did not reach a statistically significant level. Severe right ventricular dysfunction appears to be a key factor affecting these findings. In patients with posterior VSR and right ventricular infarction, VSR must be approached through the infarcted right ventricle rather than the non-infarcted left ventricle to preserve left ventricular function and to avoid inadvertent damage to a normal obtuse marginal artery/posterior left ventricular artery supplying the inferior wall of left ventricle. One should be very careful while closing the ventriculotomy as this right ventricle is extremely friable and the sutures can give way. We recommend the use of glue (cyanoacrylate adhesive) along with PTFE felt to buttress the whole inferior wall around the ventriculotomy in such patients to prevent bleeding and late aneurysm formation. The glue was applied to ventriculotomy margins before closure began in order to prevent sutures from cutting through and needle holes from bleeding. A second layer of glue can be applied later, if required, over the suture margins. We do not however recommend the routine use of glue to decrease the chance of postoperative bleeding as less postoperative bleeding and blood transfusion requirements were observed if VSR was approached through the infarcted region of left ventricle. Though in our study location of VSR did not affect results, we think apical VSRs have the best prognosis because they are mostly associated with more distal occlusion of the culprit vessel, thus preserving blood supply to most of the septum. Being small and easily accessible, they are also easier to close surgically/interventionally.

Time periods from MI to VSR and VSR to Surgery seem to be the crucial factors affecting survival. The greater chances of mortality in patients operated early after occurrence of VSR could be explained by the fact that unstable hemodynamics and ischemia-reperfusion injury to recently infarcted myocardium further stuns the myocardium. The beneficial effects of the optimum waiting period are also due to the development of firmer scars in infarcted areas of the myocardium, facilitating surgical repair, decreasing risk of recurrent or residual defects, and ultimately improving prognosis^[21]^. This was also observed in the present study, as intervals between MI and VSR and VSR and Surgery were found to be independent predictors of hospital mortality, thereby prompting their use in our scoring system. Similar observations were made by Poulsen et al.^[10]^, Papalexopoulou et al.^[22]^, and Jones et al.^[23]^. We also observed that 16 (76%) out of 21 patients died if the surgery was carried out <3 days from occurrence of VSR while only 26% (5/19) died when surgery was performed >3 days after occurrence of VSR. This suggests that, if we can delay the surgery for >3 days after the occurrence of VSR, mortality of the patients decreases by 50% and if we can delay it for 4-6 weeks, then it further decreases to almost 0. Time between VSR and Surgery appears to be the most important interval (statistically) as it signifies the time when the heart and various body systems get to adapt to the hemodynamic consequences of sudden left to right shunt. Moreover, this is the most important determinant of the quality of margins of VSR, whether fibrosed or friable. A short interval between MI and VSR is suggestive of severe ischemia and unavailability of collateral circulation. A long interval between MI and Surgery (MI to VSR + VSR to Surgery) suggests the heart is well collateralized and/or systems have had enough time to adapt to altered hemodynamics. This is where IABP proves so beneficial. We emphasize the institution of preoperative IABP to stabilize the patients. IABP acts by decreasing the afterload of left ventricle and improves coronary circulation. Ultimately, the reperfusion injury associated with cardiopulmonary bypass and revascularization is decreased, especially on a stunned myocardium. We instituted IABP in 39 patients preoperatively. One patient had severe aortoiliac disease with left subclavian stenosis, so IABP was inserted through the right subclavian artery in the operating room. Some hemodynamically unstable patients failed to stabilize despite high inotropic support and IABP could be salvaged by using ECMO. ECMO was instituted in one patient as he could not be weaned off cardiopulmonary bypass, even with IABP and higher inotropic support. ECMO could be weaned off in this patient in 60 hours and he made a complete recovery. Thus, the 'optimal delay' approach is extremely beneficial for centers without ECMO support since it improves patient outcomes.

UPMS helps in guiding the management of the patients with post-MI VSR. Based on our results, our current protocol is that, upon arrival at an emergency unit, a 'heart team' evaluates the patient. Patients with UPMS of <25 undergo emergency surgery with ECMO support, if required. Patients with UPMS of 25-75 are optimally stabilized before being taken up for surgery. This group benefits the most from 'Optimal Delay' in surgery. Patients with a score of over 75 are most likely to survive and undergo planned device or surgical closure. As described earlier, UPPS helps to prognosticate patients with post-MI VSR. All patients with UPPS of <25 died whereas none of the patients with UPPS > 75 did. Therefore, patients with a higher UPPS have higher chances of survival.

In the current study, residual ventricular septal defects were observed in 3 (7.5%) patients, which is lower than the various other series^[2,10,14,21,24]^. These defects were not present intraoperatively, but were found on postoperative echocardiography. We close most of the VSRs with interrupted mattress sutures (modified Daggett's technique^[11]^) so small jets may be present in the gaps between the sutures or due to cutting through one of the sutures. Infarct exclusion technique was used in 4 patients. However, it requires a very big patch as well as bigger ventriculotomy and patch dehiscence remains a possibility if even one bite cuts through. We feel that the interrupted suture technique is the safest bet in VSRs with friable margins, as most of the sutures will hold the patch in place, allowing for only small jets in case of suture cutting through. We also observed that the proximal margin of the defect is usually healthy as compared to the distal margin, probably due to better blood supply of proximal septum, which may have patent septal arteries and collaterals. Hence, extra care must be taken while suturing the distal margin to prevent residual ventricular septal defects. Therefore, we grafted all LADs (in case of proximal LAD or left main disease) if the first septal was visualized angiographically to preserve the blood supply and to provide better healing of ventricular septum.

### Limitation

It should be mentioned that this study had some limitations. The main limitations are the retrospective nature of our work and the sample size being smaller than some multicenter trials. Also, all late presenting VSRs (> 4 weeks) were closed by devices, which left only the worst patients for surgery. The mortality rate of our study's surgical group may thus have been fallaciously high.

## CONCLUSION

The strength of this study lies in it being a single, high-volume center data of a considerably large group of patients undergoing surgical repair of post-MI VSR, within a short time span of 5 years. Experience of a high-volume center for any less common/rare procedure provides a closer insight into the magnitude of a critical condition than multicenter trials. Messages of the study are:

1. In classical teaching, any mechanical complication of MI mandates immediate surgery. However, this study shows that patients with post-MI VSR should be stabilized first with IABP, along with inotropes and, depending upon hemodynamic patterns and UNM Post-MI VSR Management Scoring System (UPMS), the patient may either go for Immediate Surgery (score of <25), Optimal Delay (score of 25-75), or Elective Repair (score of > 75) (Figure 3). Any patient undergoing VSR closure within 3 days of its occurrence must be taken to the operation room with an integrated ECMO circuit, especially if the time interval between MI and VSR is <3 days.
2. According to the UNM Post-MI VSR Prognosis Scoring System (UPPS), a score of <25 suggests poor prognosis, 25-75 suggests intermediate prognosis, and a score of > 75 suggests good prognosis.
3. Meticulous closure of VSR with interrupted sutures taking healthy tissue in every bite gives good freedom from residual VSRs.
4. Location of VSR does not affect the outcome of the patients, but it can help in deciding the ventriculotomy site. As described earlier, posterior VSR with right ventricular infarction must be approached through RV.
5. If a patient with anterior VSR has a well-visualized first septal with left main disease or proximal significant disease of LAD, CABG with graft to proximal LAD should be performed.
6. Concomitant CABG does not affect early outcome of the patients. However, CABG may prevent deterioration of left ventricle function in patients with TVD in the long term.

**Table:** 

Authors' roles & responsibilities	
AM	Substantial contributions to the conception or design of the work; or acquisition; final approval of the version to be published
KP	Acquisition, analysis, or interpretation of data for the work; final approval of the version to be published
PS	Drafting the work or revising it critically for important intellectual content; final approval of the version to be published
VW	Revising the work critically for important intellectual content; final approval of the version to be published
TM	Interpretation of data for the work; final approval of the version to be published
JK	Final approval of the version to be published
KS	Drafting the work or revising it critically for important intellectual content; final approval of the version to be published
SP	Acquisition, analysis, or interpretation of data for the work; final approval of the version to be published
